# Disrupting the link between maltreatment and delinquency: how school, family, and community factors can be protective

**DOI:** 10.1186/s12889-019-6906-y

**Published:** 2019-05-17

**Authors:** Andra Wilkinson, Hannah Lantos, Tyler McDaniel, Hannah Winslow

**Affiliations:** 1 Child Trends, 7315 Wisconsin Ave, Suite 1200W, Bethesda, MD 20814 USA; 2 University of North Carolina Gillings School of Global Public Health, Department of Maternal and Child Health, 135 Dauer Drive, Chapel Hill, NC 27599 USA; 3 Johns Hopkins Bloomberg School of Public Health, Department of Population, Family, and Reproductive Health, 615 N. Wolfe St, Baltimore, MD 21205 USA; 4 Stanford University, Departmernt of Sociology, 450 Serra Mall, Building 120, Room 160, Stanford, CA 94303-20147 USA

**Keywords:** Maltreatment, Abuse, Delinquency, Violent, Non-violent, Protective factors, School connection, Neighborhood collective efficacy, Parental relationship quality

## Abstract

**Background:**

Past experiences of childhood maltreatment are common for youth involved in the juvenile justice system. This paper explores potential protective factors at the peer, family, school, and neighborhood levels that disrupt the relationship between maltreatment and later non-violent and violent offending behavior and how these protective effects vary by a number of different sociodemographics.

**Methods:**

We used data from the National Longitudinal Study of Adolescent to Adult Health (Add Health), a nationally representative longitudinal study of adolescents who were in grades 7–12 in the 1994–95 school year. Pulling data from Add Health respondents from ages 13 to 30, we used linear mixed effects modeling to create growth curves of predicted violent and non-violent offending frequency from adolescence into young adulthood, with maltreatment frequency as a predictor. Next, we tested whether potential protective factors including time with friends, a high-quality relationship with a parent figure, school connection, or neighborhood collective efficacy moderated the intercept or slope of the growth curves. Finally, we tested if sex, race/ethnicity, or sexual orientation moderated these protective effects.

**Results:**

For violent offending, school connection, high-quality relationships with mother or father figures, and neighborhood collective efficacy were all generally protective, meaning they were associated with lower levels and shallower slopes of predicted violent offending, but they were not more or less protective for those who experienced maltreatment. For non-violent offending, the same was true of school connection, high-quality relationships with a mother figure, and neighborhood collective efficacy, which were all generally protective. We found no evidence of a protective effect for time spent with friends, though this is likely due to measurement constraints, as simply measuring time spent with friends may have heterogeneous effects on delinquent behaviors. We found no evidence that any of these protective effects varied by sociodemographics.

**Conclusions:**

This paper identifies factors that teachers, juvenile corrections officers, policymakers and others can intervene on to prevent engagement (or re-engagement) in delinquency and offending among youth and young adults who experienced maltreatment. As they are also protective for youth who have not experienced maltreatment they also inform general delinquency prevention efforts.

## Introduction

Nine out of every thousand children (aged 0 to 17) in the United States have experienced some kind of maltreatment or abuse at the hands of a parent or caretaker [1]. The majority of this maltreatment is neglect, although children also experience physical, sexual and emotional abuse [2]. These experiences of maltreatment impact children’s well-being long past the actual instances of maltreatment. Adults’ physical [3, 4] and emotional health [5], future experiences of victimization [6], lifetime educational attainment [7], and socioeconomic stability [8] are all impacted by childhood experiences of abuse and neglect. Though most people who experience abuse or neglect as children do not go on to engage in serious delinquent behaviors [9], children who experienced maltreatment are more likely than those who did not to engage in delinquent behaviors through adolescence and into adulthood [10, 11]. Evidence for this includes the high percentage of youth involved in the juvenile justice system reporting maltreatment experiences [9].

We hypothesize in this paper that young people who experienced maltreatment and did not have certain protective factors in their lives are the ones who would be more likely to go on to engage in delinquent or criminal behavior.^1^ There are two ways that the presence of these protective factors may act to prevent negative behaviors. First, they may allow for more pro-social, healthy development to occur because youth have stronger relationships with family members, teachers, peers, or neighbors who support them. Second, youth may simply manage to avoid opportunities to engage in delinquent or criminal acts if they have more structured time, adult supervision, or pro-social friends. These two social influences on delinquency align with Hirschi’s theory of motivation versus restraint as the two sides influencing delinquent behaviors. Children with stronger social connections may have both different motivations (desire to *be* delinquent) as well as different restraints (adults and peers who discourage that behavior by their very presence) [12]. Additionally, Burgess and Akers’ 1966 theory on the learning of criminal behavior highlights the importance of contextual factors in children’s delinquency by impacting who and what they learn and the consequences they face following engagement in certain behaviors [13]. Continued, more recent, research into this theory on the part of Akers emphasizes that the more children engage with deviant peers and the more they receive benefit rather than punishment for that behavior, the less they will engage in pro-social behavior [14].

This paper models the linkage between childhood experiences of maltreatment and later offending behavior to test whether the presence of certain protective factors in a young person’s life changes the shape or magnitude of curves describing this link.

When identifying potential protective factors to explore in this paper, we focused on protective factors that were easily affected – what we called “malleable.” The malleability of a factor was important to us as we wanted to target protective factors where simple policy or programmatic changes might have an impact on children’s and young adults’ outcomes. These also seemed like the factors that program leaders working with young people day-to-day could have an impact on. For example, when examining potential protective factors, we would include the quality of family relationships but not family income, as the former could potentially benefit from an evidence-based intervention while increasing family income would likely require larger, macro-level economic changes.

In a review of the literature, we found evidence that a connection to parents, peers, school, and neighborhood potentially disrupted the link between maltreatment and offending [15, 16]. At the family level, a relationship with one’s mother or father can mediate the link between abuse and delinquency [17–21]. Additionally, friendships with peers who did not exhibit delinquent behavior can also be a protective factor for abused youth [22, 23]. School connectedness, along with peer and parent disapproval of antisocial behavior, have been found to decrease rates of lifetime violence, delinquency, and status offenses in youth exposed to physical abuse [24]. Finally, decades old sociological research has found linkages between higher levels of neighborhood collective efficacy and lower levels of neighborhood crime. This work is based on the hypothesis that neighborhood collective efficacy drive violence reduction because of social cohesion and the willingness of neighbors to intervene when problems arise [25]. More recent studies have found that shared trust and a high amount of neighborhood collective efficacy significantly reduced the odds of neglected youth, in particular, exhibiting violence during adolescence [26, 27].

While previous papers have explored the question of the link between childhood maltreatment and subsequent offending behavior, our paper adds a few important components to the analysis [28–34]. Though the existing literature provided us examples of potential protective factors, much of this research was limited in specific ways. First, while several of these studies also used Add Health data, some were limited to the first three waves or to more narrow age ranges while others did not explore protective factors [35] or explored different protective factors [36] than we did. Second, it was less common to see multiple protective factors explored together in the same study. Papers that focused on just family [37], just the school-connection piece [38], just the neighborhood [39–41], or just peers are common [42]. While these papers often do an excellent job of explaining why parental monitoring or modeling shapes youth’s behaviors, why neighborhood collective efficacy can lead to more stable or cared for neighborhoods, or how school can be a stabilizing force, youth do not exist in a vacuum. Therefore, our study sets up a way to begin to hypothesize how all of these protective factors may be important by including them all in one paper. Third, the methods of these studies were often limited. The studies were typically cross-sectional analyses, had small samples, and/or used basic regression models [43]. Fourth, many of these previous articles focus primarily on subsequent victimizing behavior and we focus on both violent and non-violent offending behavior more broadly and together in the same paper [16, 44]. Finally, they were often analyzing a homogenous population or an already at-risk population such that variation by sociodemographics could not be examined [45–47].

In this paper, we aim to discover what factors could disrupt the link between maltreatment and either delinquent or criminal behaviors. Specifically, we use linear mixed effects models to explore how self-reports of maltreatment are related to self-reports of offending in a nationally representative study sample. This method allowed us to initially define trajectories across age and time to see how maltreatment and offending behaviors were related. Now, we add in protective factors to see whether the trajectories we previously defined change with the inclusion of these protective variables. Additionally, we study whether any of the protective factors moderate this relationship differently across gender, race/ethnicity, or sexual orientation. Our ability to explore the protective factors and test for differences across sub-populations is an important addition to the literature which may allow for targeted interventions.

## Methods

### Sample

The present study used data from the National Longitudinal Study of Adolescent to Adult Health (Add Health), a longitudinal study that includes a nationally representative sample of U.S. adolescents who were in grades 7–12 in the 1994–95 school year (Wave I). There have been four in-home interviews to date. The present analysis sample is restricted to respondents interviewed at Wave I, Wave III (ages 18 to 26), and Wave IV (ages 24 to 32), with valid sampling weights at the individual, cluster, and strata levels (*N* = 12,288) and who had completed data on all variables of interest (*N* = 10,613, 86%). Low item-level missingness was confirmed before the complete case analysis and significance testing confirmed there were no significant differences between the analytic sample and the full sample. Data from Wave II were not used as Wave I seniors were not followed by design. Details of the Add Health study and design are described in other papers [48]. All Add Health procedures were approved by the Institutional Review Board at Child Trends. These analyses were deemed exempt.

### Measures

#### Independent variable: child maltreatment frequency

Childhood maltreatment was measured via a categorical variable capturing frequency (0 [never] – 10 [10 or more times]) of experiencing childhood abuse or neglect. It measured two different categories: 1) emotional, physical, or sexual abuse before age 18 or 2) supervisory neglect before sixth grade by a parent or an adult caregiver. The variable captures an additive frequency of maltreatment rather than type because recent evidence suggests the chronicity of maltreatment is a better indicator of potentially negative consequences than the type of maltreatment [49]. In our measure, an adolescent who reported experiencing emotional abuse three times before age 18 and supervisory neglect once before sixth grade would have a maltreatment frequency of four. The average maltreatment frequency in our analytic sample was 2.6 times with a standard deviation of 2.7.

#### Dependent variable: offense frequency

Delinquency and criminal offending were measured via two scales gauging frequency of different behaviors in the past 12 months. Both violent and non-violent offending, mirroring prior measures of offending using Add Health data, were included as separate variables [50, 51]. Violent offending frequency (alpha = .60–.73 across the waves) included the following indicators: shooting or stabbing someone; hurting someone badly enough to need bandages or care from a doctor or nurse; using or threatening a weapon to get something from someone; pulling a knife or gun on someone; and being in a group fight. Non-violent offending frequency (alpha = .50–.66 across the waves) included the following indicators: deliberately damaging property that didn’t belong to you; going into a house or building to steal something; stealing something worth less than $50; stealing something worth more than $50; selling marijuana or other drugs; and taking an illegal drug using a needle. The choice of indicators was constrained by what items were included in the survey, which were included in each wave; and if items fit better as control variables. Though a time-varying measure, offending frequency was not centered as the zero is conceptually meaningful and the mean would likely be little or no offending.

#### Moderator variables

Five hypothesized protective factors were tested as moderators of the relationship between maltreatment and later offending. Prior research informed the choice of potential protective factors as well as how they were measured. Potential protective factors were selected at the level of the family (relationship quality with a mother [father] figure), peers (time spent with friends), school (school connectedness), and neighborhood (neighborhood collective efficacy). **Parental relationship quality** was measured as a summative scale of five items (alpha = .95 [mothers], .98 [fathers]) that inquired about the respondent’s relationship with a parent or parental figure: how close do you feel to your mother/father, how much do you think she/he cares about you, most of the time your mother/father is warm and loving towards you, you are satisfied with the way you and your mother/father communicate, and overall you are satisfied with your relationship with your mother/father [52–54]. The scales were created separately for mothers and fathers as the scales were not highly correlated. Respondents were coded as ‘0’ in either scale if they reported not having a relationship with a mother or father figure. Respondents were classified as having high-quality parental relationships if the scale score was around the mean or higher. The mean was selected as the scale was skewed positively and a value between the means of the scale for mother and father figures was chosen to use the same cut point for both scales. **Time with friends** was measured with a single item assessing how many times the respondent had hung out with friends in the past week (0 [not at all] – 3 [5 or more times]). **School connectedness** was measured with a standardized summative scale (alpha = .73) of eight items assessing if the respondent feels like they are a part of their school, close to the people at school, feel safe at school, feel the teachers care about them, etc. [55, 56] **Neighborhood collective efficacy** was measured with a standardized summative scale of five items (alpha = .60) estimating whether the respondent feels safe in their neighborhood, thinks people in the neighborhood look out for each other, communication between neighbors, knowing most of your neighbors, and satisfaction living in the neighborhood [57]. Both the standardized scales were tested comparing mean to high scores (two standard deviations above the mean) and low (two standard deviations below the mean) to high scores, for sensitivity analyses.

#### Control variables

Previously published relevant analyses were reviewed to inform the potential confounders that should be controlled for [58–61]. Individual characteristics specifically tested for in the models included sex, race/ethnicity from Wave I (Hispanic and non-Hispanic White, Black, Asian, Native American, and Other), and sexual orientation/attraction (respondent was categorized as non-heterosexual if they identified as homosexual or bisexual or if they reported attraction to the same sex). Trouble in school was measured with an indicator of whether respondent had ever repeated or been held back a grade, while another indicator assessed if they had ever been suspended, expelled, or dropped out. An indicator of whether anyone in the household had received public assistance before the respondent was 18-years-old was used to approximate the socioeconomic status of their childhood home. Whether the respondent had ever lived in a foster home was also included. Finally, any use of substances before Wave I was controlled for; the substances included alcohol, cigarettes, and marijuana. Other illicit drugs and injection drug use were not included in this measure as injection drug use was included in the non-violent offending frequency measure.

### Analyses

The dataset was structured by age instead of wave to capture the trajectory from adolescence to young adulthood. Linear mixed effects models were used to estimate growth curves of the two dependent variables: violent and non-violent offending. Forty models were fit for each of the two dependent variables. The first ten models were used to test moderation of the relationship between childhood maltreatment and the intercept and slope of offending frequency by five potential protective factors. Two- and three-way interaction terms were used to test the potential protective factors. The next thirty models tested whether the moderating effect of the potential protective factors varied by gender, race/ethnicity, and sexual orientation. Three- and four-way interaction terms were used to test variation in the potential protective effects by sociodemographics. These models were tested as moderation models rather than mediation ones because the linear mixed effects models tested the association between childhood maltreatment and then offending frequency reported at three junctures over time. By doing this, the models produce a curve rather than an association and so lend themselves to moderation analysis rather than mediation analysis to test if anything “bends the curve.”

All significant models were run with a random intercept and slope to examine variation in the effect. The intraclass correlation coefficient (ICC), used in linear mixed effects models to determine the percentage of variance in offense frequency that is due to variance between individuals, was used in these analyses. However, the sampling weights for analyzing the Add Health data inhibit testing if the ICC is significantly different than zero. So, the ICC from the first, basic model without covariates, and a model with a protective factor were compared to determine how much of the variance in offense frequency was explained by the predictor variables.

## Results

Approximately half of the analytic sample (Table 1) were female, and half were male. More than one third of the sample were young people of color. Over one tenth of the sample (12.2%) reported a sexual orientation other than heterosexual. The majority of the sample (77.0%) reported experiencing at least one type of childhood maltreatment. During their teen years in Wave I, nearly one third of the sample had engaged in non-violent offending behavior (32.7%), and three in ten had committed at least one violent offending behavior (30%).

**Table 1:** Descriptive data on analytic sample

	N or mean	Weighted % or STD
Sex		
Male	5373	50.6%
Female	5240	49.4%
Race/ethnicity		
Hispanic	1249	11.8%
Black	1600	15.1%
Asian	375	3.5%
Native American	217	2.0%
Other	102	1.0%
White	7070	66.6%
Sexual Orientation		
LGBQ	1305	12.3%
Age at Wave I	15.4	1.8
Age at Wave III	21.8	1.9
Age at Wave IV	28.3	1.9
Control variables		
Public assistance in household before age 18	1673	15.8%
Ever repeated or been held back a grade	2150	20.3%
Ever suspended, expelled or dropped out	142	1.3%
Ever used alcohol, cigarettes, or marijuana	6033	58.1%
Ever in a foster home	173	1.6%
Moderators		
School connectedness scale (standardized)	0.0	1.0
Mom relationship quality scale (0–25)	21.0	5.8
Dad relationship quality scale (0–25)	15.6	9.9
Neighborhood collective efficacy Scale (standardized)	0.0	1.0
Number of times hung out with friends in past week		
Not at all	950	8.6%
1 to 2 times	2536	23.4%
3 or 4 times	2886	26.8%
5 or more times	4241	41.1%

Maltreatment frequency varied across demographics (in adolescence) (Table 2). The average maltreatment frequency in a given year was highest for Native Americans (M = 3.56, SD = 3.18), and lowest for Whites (M = 2.54, SD = 2.40).

**Table 2:** Sociodemographic variation in frequency of maltreatment, non-violent and violent offenses in adolescence

	Average maltreatment frequency		Average non-violent offense frequency in adolescence		Average violent offense frequency in adolescence	
	Mean	Std. Dev.	Mean	Std. Dev.	Mean	Std. Dev.
Sex						
Male	2.51	2.48	1.13***	1.93	0.97***	1.87
Female	2.78***	2.92	0.58	1.47	0.45	1.28
Race/ethnicity (white = referent)						
Hispanic	2.88**	3.22	1.07*	2.33	1.13***	2.74
Black	2.64	3.10	0.65*	1.78	1.01***	2.28
Asian	3.31***	4.03	0.97	2.65	0.63	2.06
Native American	3.56***	3.18	1.26*	2.05	1.26***	2.11
Other	2.52	2.12	1.09	2.11	0.58	1.62
White	2.54	2.40	0.84	1.58	0.57	1.24
LGBQ						
No	2.53	2.65	0.82	1.74	0.72	1.67
Yes	3.40***	2.93	1.10***	1.99	0.67	1.59

*p-value< 0.05; **p-value< 0.01; and ***p-value< 0.001 in post hoc testing

The results of these analyses can be broken down into two main parts: first, we studied whether potential protective factors moderated the association between maltreatment and offense frequency, then we studied whether the moderation by the protective factors varied by sociodemographics. In the first part, we found that school connectedness, a quality maternal/paternal relationship, and neighborhood collective efficacy did not significantly moderate the association between maltreatment and violent and non-violent offense frequency, though they still have a protective effect. In the second part, we found little evidence that sociodemographics change the moderation by the protective factor of the relationship between maltreatment and offense frequency.

### School connectedness

**Fig. 1:**
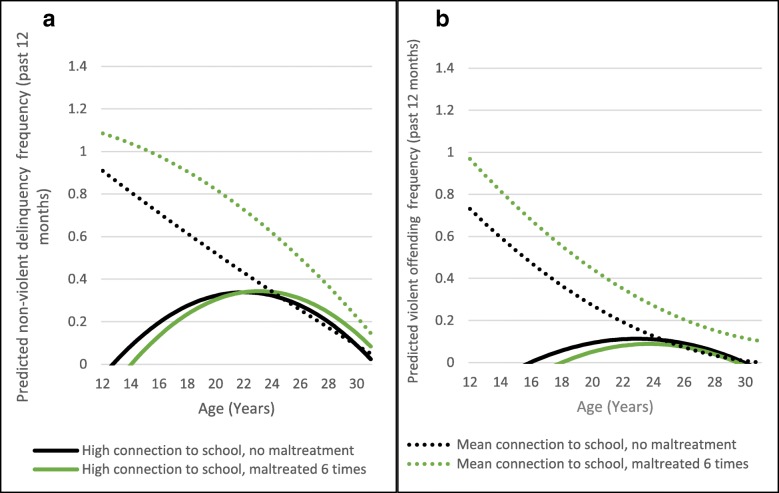
**a** Moderation of Non-violent Offending Frequency by School Connectedness. **b** Moderation of Violent Offending Frequency by School Connectedness

Connection to school appears to moderate both non-violent (Fig. 1a) and violent (Fig. 1b) offending frequency.

A high connection to school, compared to an average connection to school, significantly changed the slope and intercept of predicted non-violent and violent offending (Fig. 1a and b). For both the effect is largest in magnitude in adolescence, with approximately a one incidence of delinquency gap in predicted offending frequency for those with average compared to high connection to school. The gap decreases in magnitude across adolescence and appears to expire in the early 20s for those who did not experience maltreatment; the protective effect appears to last longer into the late 20s for those who did experience maltreatment. For non-violent offending frequency, a high connection to school makes predicted offending frequency very shallow across development. For violent offending, a high connection to school makes predicted offending nearly zero across early adolescence for both those who had and had not experienced maltreatment. Testing the same models comparing a high connection to school to a low, rather than average, connection produced results that continued the pattern as we expected; those with lower connection to school had even worse predicted outcomes.

### Parental figure relationship quality

**Fig. 2:**
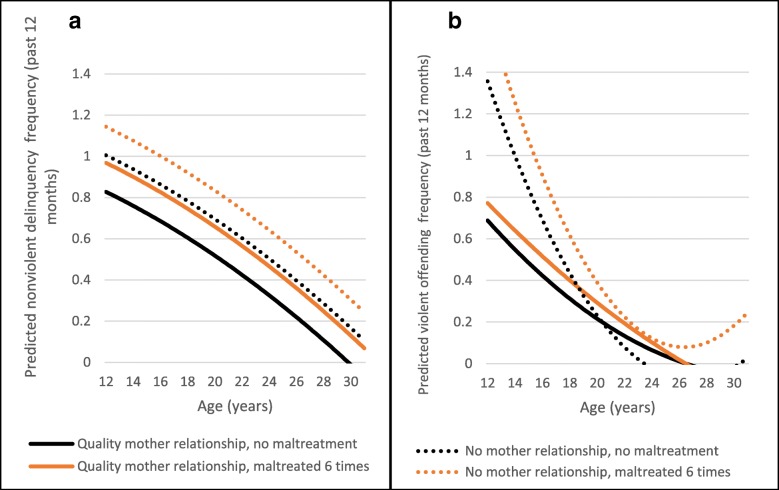
**a** Moderation of Non-violent Offending Frequency by Mother Relationship. **b** Moderation of Violent Offending Frequency by Mother Relationship

A high-quality relationship with a mother figure negatively moderated both predicted violent and non-violent offending frequency (Fig. 2a and b). Specifically, a high-quality maternal relationship appeared to moderate the intercept of predicted non-violent offending frequency, decreasing the predicted offending frequency starting in adolescence for both those who had experienced maltreatment and those who had not (Fig. 2a). For violent offending frequency, quality maternal relationships appeared to moderate both the intercept and slope of predicted violent offending frequency. The gap was present in adolescence, but the effect faded by early adulthood. There was no evidence this relationship varied by maltreatment status, suggesting that a relationship with a maternal figure is protective for all adolescents.

**Fig. 3:**
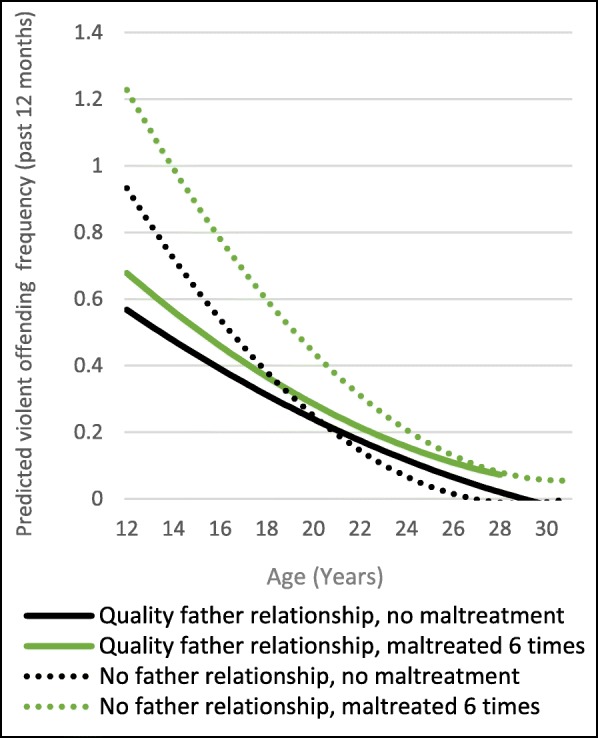
Moderation of Violent Offending Frequency by Father Relationship

A high-quality relationship with a father figure is also associated with declines in violent offending. Compared to those with no quality father relationship, those with a high-quality father relationship had significantly lower predicted violent offending frequencies in adolescence (Fig. 3). However, this association also does not vary by maltreatment frequency.

### Neighborhood collective efficacy

**Fig. 4:**
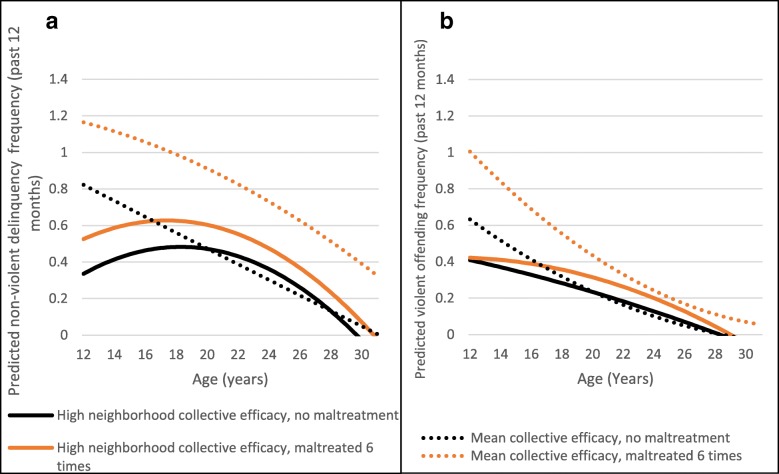
**a** Moderation of Non-violent Offending Frequency by Neighborhood Collective Efficacy. **b** Moderation of Violent Offending Frequency by Neighborhood Collective Efficacy

Neighborhood collective efficacy significantly moderated the intercept and slope of predicted non-violent and violent offending frequency. For non-violent criminal behavior, neighborhood collective efficacy appeared to decrease predicted offending, these effects persisted across development for those who experienced maltreatment and disappeared in emerging adulthood for those who did not experience maltreatment. Note that the shapes of these curves are different, suggesting that the pattern of delinquent behaviors is different for those with high versus average neighborhood collective efficacy, but the protective effect remains the same (distance between dotted and solid lines at the intercept). We also found no evidence that this relationship varied by maltreatment status (Fig. 4a). High neighborhood collective efficacy, compared to average collective efficacy, significantly lowered the intercept and decreased the slope of predicted violent offending frequency, though the protective effect did not last as long for violent offending as it did for non-violent offending. Once again, there was no evidence of variation by maltreatment status (Fig. 4b). Testing the same models comparing high collective efficacy to low, rather than average, produced similar patterns that moved in the expected directions.

### Variation by sociodemographics

In the final part of our analysis, we examined sociodemographic variation in protective factors, specifically looking for disparities in the effect of a protective factor on the relationship between maltreatment and offense frequency. We found two types of results. First, sex does significantly moderate the protective effect of school connection on the relationship between maltreatment and violent offending such that it appears more protective for females compared to males. This figure is not shown because when the predicted lines were plotted, it became clear school connection is really only more protective for females who have not experienced maltreatment. Among those who had experienced maltreatment, school connection was equally protective of violent offending for both males and females. Our second result was that sociodemographic variables moderated the association between the protective factors and offense frequency, but did not appear to moderate the protective factors’ effect on the relationship between *maltreatment* and offense frequency. For example, though the relationship between father relationship quality and offending frequency varies by race/ethnicity, father relationship quality did not change the relationship between maltreatment and offending frequency across race/ethnicity. Because the sociodemographic variation in the protective factors was unrelated to maltreatment status, these results are outside the scope of our research questions, and we do not cover these findings in more detail.

Comparing the ICCs across the respective models (Table 3), we noted decreases in the ICC, indicating the predictor variables (protective factors) did explain some of the variance in offending frequency. For example, the ICC baseline model for violent offending frequency indicates 16% of the variance in violent offending frequency is due to variance between individuals. When school connection was added in, the ICC was reduced to 11%, meaning the bulk of the variance in violent offending is within individuals over time, rather than between them. The ICC for non-violent offending models also decreased from 19 to 13% with school connection. Random effects by intercept and slope did not add meaningful variation to any of the demonstrated models.

**Table 3:** Intraclass correlation coefficients

Violent offending		Non-violent offending	
M1: Baseline model	M12: School connection protective factor	M1: Baseline model	M12: School connection protective factor
ICC = 0.16	ICC = 0.11	ICC = 0.19	ICC = 0.13

## Discussion

In this paper, our key research question focused on whether potential protective factors interrupted the link between maltreatment and later offending behavior for adolescents and young adults. Specifically, we tested whether the presence of certain potential protective factors in adolescents’ lives changed either the direction (slope) of this relationship across development or the level (intercept) of it. We found that protective factors do change the trajectory of offending behavior for youth. We were particularly interested in protective factors across multiple levels [62] that may have actionable next steps for policymakers and practitioners. Each of the factors we chose is supported by literature as protective against engagement in delinquent behaviors. Specifically, we included peer relationships, relationship quality with a mother and/or father figure [63, 64], connection to school [60], and neighborhood collective efficacy [65].

There are two patterns of results that we found in these models. In order from strongest to weakest evidence of the protective effect, we found moderation of predicted offending behavior in terms of both intercept and slope and moderation in terms of just intercept. We did not find moderation of the association between maltreatment and offending behavior. Put another way, the protective factors were as effective for youth who experienced maltreatment as youth who had not. Further, in some instances, the predicted protective effect appeared to last longer into development for youth who experienced maltreatment, compared to those who had not. So, it may not be more protective in magnitude but could be more protective in longevity. We discuss each of these in more detail below.

Figure 1 demonstrates school connectedness has a protective effect for both non-violent and violent behavior. Those who had a high connection to school have an intercept that starts relatively low, and offending behavior increases during early adolescence, followed by a predicted decrease in young adulthood. The predicted increase in offending starts earlier for non-violent compared to violent offending. Those who had a mean connection to school have a high intercept in adolescence that steadily declines into young adulthood. That the protective effect for those who had not experienced maltreatment ends in the early 20s makes sense as most students have either graduated or left the school they were connected to. Interestingly, the protective effect seems to last until the early 30s for youth who experienced maltreatment. This finding deserves further exploration in future analyses. It is also key to note here that we compared high to average school connection. We felt that it was most important to describe the patterns experienced by the majority of adolescents (rather than the extremes) but, as would be expected, we see even worse outcomes for those with low school connection (results not shown).

Children spend a significant portion of their lives in school, and school connection may be particularly protective against offending behavior because it is reliable and consistent: for most adolescents, attending school is a regular part of their lives. Connection to school may also mean that youth are more likely to be engaged in supervised activities at school that keep them out of trouble, or that they are more likely have relationships with teachers or administrators who can be role models and reinforce more pro-social behaviors [66, 67]. For school connection, we might also expect to see (and, in fact, we do see) the protective effects of this factor weaken once adolescents have graduated from high school. The positive influence of this factor on delinquent behaviors during the adolescent years diminishes over time. Previous analyses have found similar patterns [68]. Our analyses also found the protective effect weakened faster for youth who did not experience maltreatment, compared to those who had suggesting perhaps that school connection is particularly important for youth who may have experienced more difficult adult relationships in other settings. However, this weakening over time may also have an additional analytic explanation: protective factors were measured only at Wave I. With continued measurement of these variables (especially for adolescents who matriculated to college), we could test if school connection in secondary education is also protective, and whether is more or less protective for youth who experienced maltreatment. This finding could help distill whether the fade in the protective effect in emerging adulthood for youth who were not maltreated is developmentally driven – when offending naturally decreases – regardless of secondary education, or if continued schooling continues to be protective.

The second pattern seen in these results is that there is evidence of moderation of offending frequency for the intercept only. Figure 2a is a good example of this pattern. This graph has lines that are parallel but with different intercepts. In this model, we see a quality relationship with a mother figure is protective of Non-violent offending frequency, both for those who experienced maltreatment and those who had not. Figs. 2b and 3 show quality parental relationships are generally protective for violent offending as well, with no apparent differences between relationships with mother or father figures. For non-violent offending, quality relationships with father figures were not protective, by a close margin and with a similar pattern over time as for mother figures. We tested the effect of strong mother and father relationships separately here because they were not correlated, but it is important to note that the results were very similar: strong parental figure relationships matter.

Maltreatment and abuse are experiences that impact a child or adolescents’ ability to cope effectively with stress which can, in turn, lead to acting out. Trusting, safe relationships can often be utilized to teach and develop coping skills [69]. Children who experience maltreatment are often hurt by the people who are supposed to care about them the most: their parents, other family members, or other adults they interact with. For this reason, it may be particularly important for children who have had these negative experiences to have strong supporting adult relationships [70] – and for most children, their strongest and most reliable relationships are with their parents. This may not be true if a parent is the perpetrator of the abuse, but in that case, the other parent could potentially be a source of support. It is also important to note two methodological considerations we made in these models. We compared high quality relationships with no relationships, as adolescents with no relationships are often not included in low/high comparisons, leaving out the potentially most vulnerable group. Also, we ran the models for maternal and paternal relationships separately because having a high-quality relationship with one parent was not correlated with a high-quality relationship with the other. Yet, we found almost identical results, meaning the effect of a high-quality parental relationship does not vary by whether it is with a mother or father. This means that one parent could have been the perpetrator for some of these respondents, but a strong relationship with the other parent may *still* be protective, especially for violent offending.

The finding that some of the protective factors are protective regardless of maltreatment status is an encouraging finding: these protective factors can have positive impacts on all children relating to a specific outcome [71]. Notably this protective effect was present for both violent and non-violent offending. This is particularly important as we know that maltreatment can be hard to detect and that identifying kids (or asking that they self-identify) can be traumatic or upsetting for some. Thus, while identifying experiences of maltreatment is incredibly important to both address ongoing maltreatment and prevent offending, focusing on the presence of strong, protective factors for all adolescents can help prevent offending.

In addition to these two patterns that described the results overall, we also saw that there were some different patterns between the non-violent and violent results. This pattern has been found elsewhere in the literature, some noting that while most offenders have poor inhibition skills, violent offenders are particularly bad and also that violent offenders tend to struggle with worse mental health issues [72, 73]. Seeing the differences across the two types of offending behaviors suggests that protective effects may work differently across them. Also, seeing the same pattern across the violence outcomes in these four graphs suggests there may be a more similar mechanism at work for violent behaviors. One potential is that violence is less common, and the adolescents or young adults who engage in violent behavior are likely different than those who engage in non-violent behaviors. A second underlying difference between violent and non-violent behaviors is that the harm that a violent adolescent is doing to another person may be more obvious. For violent behaviors, the young person sees the person they are hurting in front of them while with non-violent behaviors, the perpetrator may not be known by the victim such that some of these behaviors (i.e., theft) may be more anonymous. There is some evidence that perpetrators of violent crime are more likely to struggle with emotion regulation, social isolation, and aggression [74], and, as mentioned above, inhibition. The protective factors that we have chosen may specifically focus on human connection and empathy (school connection, parental relationships, and potentially collective efficacy to some extent, rely on connections to other humans). Thus, they may be associated with reductions in those behaviors in different ways. In this way, their relationship to violent behaviors may be very different than their relationship to non-violent behaviors. Given the previous literature, a focus on executive functioning skills and mental health treatment may be particularly important for violent offenders. While we are not able to test for these differences, we suggest it might be worth exploring in future research.

An example of the underlying mechanism preventing both violent and non-violent outcomes for all adolescents being potentially the same was for neighborhood collective efficacy. For both non-violent and violent outcomes, neighborhood collective efficacy was generally protective. Neighborhood collective efficacy likely impacts behavior through two channels: opportunity to engage in certain behaviors and watchful adults [25]. First, a higher level of neighborhood collective efficacy is associated with less violence overall [25]. It is important to note that this is true regardless of neighborhood income level – even lower income neighborhoods with high levels of collective efficacy have less violence [75, 76]. Therefore, both adolescents who were maltreated and those who were not may have fewer opportunities to engage in both non-violent and violent behaviors in these neighborhoods. Second, Fagan et al.’s conclusions about the mechanisms behind why neighborhood collective efficacy matters provide insight about its importance, why it might be the same for both types of behavior, and specific interventions that programs could focus on. They find that children in neighborhoods with high collective efficacy know two things: that they are more likely to be supervised by adults and that there are more adults around them to support them when needed, both factors that result in them being less likely to engage in criminal behaviors [65]. Programmatic interventions may want to focus on this type of neighborhood collective action if they want to utilize the collective efficacy protective factor specifically. For instance, while there has been no academic evaluation of the work that Mothers Against Senseless Killings (MASK) does in Chicago, one can easily see that their model of setting up chairs on street corners in the summer while watching and providing food for children is related to Fagan et al’s proposed mechanisms. According to city statistics, MASK appears to be reducing violent behaviors in the neighborhood where mothers watch and care for children [77].

Finally, we had two sets of important null findings. We did not see any significant patterns in the models that included time spent with friends. We hypothesized that this measure might be a good indicator of connection to peers and social connection that would be associated with less engagement in delinquent behaviors [23]; however, given these null results, we expect that time spent with friends could be either a negative or a positive. This is not a surprising finding given that who youth spend time with and how that impacts their behavior is a complex, nuanced process. How important that peer is, whether they are a best friend or not, and how long the young person has known them all appear to matter [78, 79]. Additionally, whether friends influence delinquent behavior or children who are more inclined to delinquent behavior find “like” others is challenging to disentangle [59, 80, 81]. Therefore, a measure that better differentiates the type of friends and could separate positive versus negative influence would be stronger. For example, spending time with friends at a school sports practice may be beneficial, while spending time with friends getting into trouble is, clearly, not. We will discuss this more in the limitations below as we think that the variables we used could be strengthened in future research. We also saw that there were no differences in how the protective factors changed the shape or height of the relationship between maltreatment and offending by any of the sociodemographic variables we included (sex, race/ethnicity, or sexual orientation). This is, again, affirming to us. Protective factors are important for all children; it is not just protective for specific groups, for instance black children or females. Rather all children – including those who were not maltreated – see a protective effect of these supportive, pro-social factors. This is important because it means that we can focus on providing these services to all children across the board.

There are a number of strengths to these analyses. We used a nationally representative, large, diverse, and longitudinal sample. We also were able to include and study protective factors at multiple levels – something many papers are unable to do as their data source is more focused. Most other datasets also do not have robust data on different sub-populations and for populations that might be quite small. However, with this data, we were still able to test for differences across numerous sub-populations. We were also able to compare multiple response categories as opposed to simply comparing binary “yes/no” categories. As a result, we could compare high scores on these scales to mean scores. This is a more conservative estimate than comparing to “low” levels as, by definition, there are more children around the mean, and therefore we are comparing to the “norm.” Also, linear mixed effects models allowed for nuanced testing of relationships with better controls for endogeneity. We were able to report on relationship in the intercept and slope and examine change across development rather than just a significant association or not. Additionally, by adjusting for time invariant unobserved characteristics, the models are less vulnerable to endogeneity.

These models are not without limitations. First, our measure of social connection was quite limited. The peer support measure had almost no variation which limited us to a measure of time spent with friends. However, time spent with friends can be a positive indicator – the child has friends, is close to friends, is pro-social – or can be a negative indicator – they spend time with friends who are a negative influence [82]. Our lack of significant findings at this level raise questions for us about whether our measure perhaps captured some of both these positive and negative influences – either for different adolescents or even within the same adolescent. Unfortunately measures of peer delinquency were not available at the waves we needed them, they could have improved our measure of social connection. Second, our use of an age squared term in the models allowed for nonlinearity in the predicted offending frequencies over time but also only allowed for one curve in the lines.. We tested some models adding an age cubed term to see if it improved the interpretability of the simple slopes, but the terms were not significant.

These models inform several potential future analyses. We chose protective factors that were supported by the literature, but there are likely multiple protective factors at each level that matter. Additionally, these factors are not protective in a vacuum and likely affect one another. Including multiple protective factors in the same model may be an important next step. Also, including information about who the perpetrator of the maltreatment was would add nuance – especially for the parent relationship models if the perpetrator was a parent. We also see evidence of the protective factors fading over time and at different rates for those who were maltreated and those who were not. This fading could be conceptual or analytical: there are explanations for why the mechanism of school connection or neighborhood collective efficacy might lessen over time, but we also only measured these protective factors at Wave I. Specifically, being in school or in one’s neighborhood changes as one gets older, but continued measurement of these factors may show less fading over time if an adult continues on to college or stays in the same neighborhood. This also relates to unanswered questions about how the timing of both maltreatment and the presence of the protective factor are related, which remains unclear. We only know that at Wave I, respondents reported an experience of maltreatment in childhood and reported their present levels of protective factors. So, we have moderate confidence the protective factors happened after the maltreatment, but more detail on the timing of and length of time between maltreatment and the protective factor as it relates to offending could prove even more informative. Finally, we also do not often know *how* these factors matter. What the mechanism is that these factors support to reduce anti-social behaviors and offending deserves more exploration. We have included some hypotheses in this discussion, but they deserve explicit testing moving forward. These remaining research questions should guide future work so that information about the most essential protective factors can inform efforts towards both the prevention of first-time offending and the reduction of recidivism.

## Conclusions

Overall, these analyses in a nationally representative sample indicate engagement in school, quality relationships with mother or father figures, and a sense of neighborhood collective efficacy are protective against violent and non-violent offending behavior. This was true both for youth who experienced maltreatment and those who did not, though the protective effect may last longer into development for youth who experienced maltreatment. There was no evidence that time spent with friends is protective, likely because the measure was inadequate as the type of friend (e.g., closeness) and the friend’s behavior (e.g., prosocial or not) need to be considered. Importantly, we found no variation in these protective effects by sex, race/ethnicity, or sexual orientation, indicating the protective factors matter for all youth, not just certain youth.

These results have implications for dual system youth – youth involved in both the child welfare and juvenile justice systems—who tend to fare worse than those involved in either system. These results help the workers in both systems target their limited resources (e.g., by focusing on increasing supports for a youth missing many protective factors compared to a youth who already has a strong connection to their school). Increasing protective factors for youth who need them most could prevent offending behavior, prevent recidivism for youth already involved in the justice system, foster positive youth development, increase public safety and decrease public costs.

## Abbreviations

Add Health: National Longitudinal Study of Adolescent to Adult Health
ICC: Intraclass correlation coefficient
MASK: Mothers against senseless killings

## Footnotes

1 Delinquency is the word used to describe behaviors for children under the age of 18. Once a child becomes an adult, these behaviors are simply referred to as criminal. As the present study examines criminal activity from adolescence into young adulthood, we refer to these as “offenses” or “criminal offenses” and the behavior as offending in order to capture behaviors both before and after a respondent turned 18.

## References

[1] Centers for Disease Control and Prevention. Child abuse and neglect: Definitions. 2016. https://www.cdc.gov/violenceprevention/childabuseandneglect/index.html. Accessed 14 Feb 2018.

[2] U.S. Department of Health and Human Services, Administration for Children and Families, Children’s bureau. Child maltreatment 2016; 2018. https://www.acf.hhs.gov/sites/default/files/cb/cm2016.pdf.

[3] Archer G, Pinto Pereira S, Power C. Child maltreatment as a predictor of adult physical functioning in a prospective British birth cohort. BMJ Open. 2017. 10.1136/bmjopen-2017-017900. 10.1136/bmjopen-2017-017900 PMC5665268 29079607

[4] Sacks R, Takemoto E, Andrea S (2017). Childhood maltreatment and BMI trajectory: the mediating role of depression. Am J Prev Med.

[5] Norman R, Byambaa M, De R, Butchart A, Scott J, Vos T (2012). The long-term health consequences of child physical abuse, emotional abuse, and neglect: a systematic review and meta-analysis. PLoS Med.

[6] Tillyer MS (2015). The relationship between childhood maltreatment and adolescent violent victimization. Crime Delinq.

[7] Hardner K, Wolf M, Rinfrette E. Examining the relationship between higher educational attainment, trauma symptoms, and internalizing behaviors in child sexual abuse survivors. Child Abuse Negl. 2017. 10.1016/j.chiabu.2017.10.007. 10.1016/j.chiabu.2017.10.007 29074261

[8] Snehal M, Pereira P, Li L, Power C (2017). Child maltreatment and adult living standards at 50 years. Pediatrics..

[9] Goodkind S, Shook JJ, Kim KH, Pohlig RT, Herring DJ (2013). From child welfare to juvenile justice: race, gender, and system experiences. Youth Violence Juv Justice.

[10] Mersky J, Topitzes J, Reynolds A. Unsafe at any age: linking childhood and adolescent maltreatment to delinquency and crime. J Res Crime Delinq. 2012;49(2):295–318 https://www.ncbi.nlm.nih.gov/pmc/articles/PMC5115874/. 10.1177/0022427811415284 PMC5115874 27867220

[11] Currie J, Tekin E (2012). Understanding the cycle: childhood maltreatment and future crime. J Hum Resour.

[12] Hirschi T (1995). Causes and prevention of juvenile delinquency. Contemporary masters in criminology.

[13] Burgess RL, Akers RL (1966). A differential association-reinforcement theory of criminal behavior. Soc Probl.

[14] Akers RL. Social Learning and Social Structure : A General Theory of Crime and Deviance. Boston: Northeastern University Press; 1998. https://catalyst.library.jhu.edu/catalog/bib_1998754. Accessed 27 Jan 2019.

[15] Hawkins JD, Weis JG (1985). The social development model: an integrated approach to delinquency prevention. J Prim Prev.

[16] Kim E, Gilman A, Hill K, Hawkins J (2016). Examining protective factors against violence among high-risk youth: findings from the Seattle social development project. J Crim Justice.

[17] King V, Boyd LM, Pragg B (2018). Parent–adolescent closeness, family belonging, and adolescent well-being across family structures. J Fam Issues.

[18] Craig JM (2016). Which bond matters more? Assessing the differential strengths of parental bonding measures on adolescent delinquency over time. Youth Violence Juv Justice..

[19] Harris-McKoy D, Cui M (2013). Parental control, adolescent delinquency, and young adult criminal behavior. J Child Fam Stud.

[20] Meadows SO (2007). Evidence of parallel pathways: gender similarity in the impact of social support on adolescent depression and delinquency. Soc Forces.

[21] Warr M (2007). The tangled web: delinquency, deception, and parental attachment. J Youth Adolesc.

[22] Salzinger S, Rosario M, Feldman R (2007). Physical child abuse and adolescent violent delinquency: the mediating and moderation roles of personal relationships. Child Maltreat..

[23] Reynolds AD, Crea TM (2015). Peer influence processes for youth delinquency and depression. J Adolesc.

[24] Herrenkohl TI, Tajima EA, Whitney SD, Huang B. Protection against antisocial behavior in children exposed to physically abusive discipline. J Adolesc Health. 2005. 10.1016/j.jadohealth.2003.09.025. 10.1016/j.jadohealth.2003.09.025 15901510

[25] Sampson RJ, Raudenbush SW, Earls F (1997). Neighborhoods and violent crime: a multilevel study of collective efficacy. Science (80- ).

[26] Yonas MA, Lewis T, Hussey JM (2010). Perceptions of neighborhood collective efficacy moderate the impact of maltreatment on aggression. Child Maltreat.

[27] Deutsch AR, Crockett LJ, Wolff JM, Russell ST (2012). Parent and peer pathways to adolescent delinquency: variations by ethnicity and neighborhood context. J Youth Adolesc..

[28] Watts SJ (2017). The link between child abuse and neglect and delinquency: examining the mediating role of social bonds. Vict Offender.

[29] Yun I, Ball JD, Hyeyoung Lim H (2011). Disentangling the relationship between child maltreatment and violent delinquency: using a nationally representative sample. J Interpers Violence.

[30] Wiig J, Spatz Widom C, Tuell J. Understanding Child Maltreatment & Juvenile Delinquency. Washington, DC; 2003. https://rfknrcjj.org/images/PDFs/Understanding_Child_Maltreatment_and_Juvenile_Delinquency_From_Research_to_Effective_Program_Practice_and_Systemic_Solutions.pdf

[31] Savage J, Palmer JE, Martin AB (2014). Intergenerational transmission: physical abuse and violent vs. non-violent criminal outcomes. J Fam Violence.

[32] Watts SJ, McNulty TL (2013). Childhood abuse and criminal behavior: testing a general strain theory model. J Interpers Violence..

[33] Jang SJ, Rhodes JR (2012). General strain and non-strain theories: a study of crime in emerging adulthood. J Crim Justice..

[34] Turanovic JJ, Pratt TC (2015). Longitudinal effects of violent victimization during adolescence on adverse outcomes in adulthood: a focus on prosocial attachments. J Pediatr.

[35] Watts SJ, Iratzoqui A (2019). Gender, child maltreatment, and delinquency. Vict Offender..

[36] Bunch JM, Iratzoqui A, Watts SJ (2018). Child abuse, self-control, and delinquency: a general strain perspective. J Crim Justice..

[37] Valgardson BA, Schwartz JA (2019). An examination of within- and between-family influences on the intergenerational transmission of violence and maltreatment. J Contemp Crim Justice.

[38] Elise Barboza G, Siller LA. Child maltreatment, school bonds, and adult violence: a serial mediation model. J Interpers Violence. 2018:1–35. 10.1177/0886260518805763. 10.1177/0886260518805763 30392439

[39] Sampson RJ (2008). Moving to inequality: neighborhood effects and experiments meet social structure. Am J Sociol.

[40] Kling J, Ludwig J, Katz L. Neighborhood effects on crime for female and male youth: evidence from a randomized housing mobility experiment. Q J Econ 2005;120:87–130. https://scholar.harvard.edu/lkatz/publications/neighborhood-effects-crime-female-and-male-youth-evidence-randomized-housing-mobi. Accessed 30 Jan 2019.

[41] Ludwig J, Duncan G, Gennetian L (2013). Long-term neighborhood effects on low-income families: evidence from moving to opportunity. Am Econ Rev Pap Proc.

[42] Bender K (2010). Why do some maltreated youth become juvenile offenders? A call for further investigation and adaptation of youth services. Child Youth Serv Rev.

[43] Gold J, Wolan Sullivan M, Lewis M (2011). The relation between abuse and violent delinquency: the conversion of shame to blame in juvenile offenders. Child Abuse Negl.

[44] Richards TN, Tillyer MS, Wright EM (2017). Intimate partner violence and the overlap of perpetration and victimization: considering the influence of physical, sexual, and emotional abuse in childhood. Child Abuse Negl.

[45] Herrenkohl RC, Egolf BP, Herrenkohl EC (1997). Preschool antecedents of adolescent assaultive behavior: a longitudinal study. Am J Orthop.

[46] Smith C, Thornberry TP (1995). The relationship between childhood maltreatment and adolescent involvement in delinquency. Criminology..

[47] Loeber R, Pardini D, Homish D (2005). The prediction of violence and homicide in young men. J Consult Clin Psychol.

[48] Harris K. The Add Health study: design and accomplishments. http://www.cpc.unc.edu/projects/addhealth/data/guides/DesignPaperWIIV.pdf. Published 2013. Accessed 22 Mar 2016.

[49] Font SA, Berger LM (2015). Child maltreatment and children’s developmental trajectories in early to middle childhood. Child Dev.

[50] Guo G, Roettger ME, Shih JC. Contributions of the DAT1 and DRD2 genes to serious and violent delinquency among adolescents and young adults. Hum Genet. 2007. 10.1007/s00439-006-0244-8. 10.1007/s00439-006-0244-8 17120049

[51] Beaver K, Connolly E, Schwartz J, Boutwell B, Barnes J, Nedelec J (2016). Sexual orientation and involvement in non-violent and violent delinquent behaviors: findings from the national longitudinal study of adolescent to adult health. Arch Sex Behav.

[52] Pearson J, Muller C, Frisco M (2006). Parental involvement, family structure, and adolescent sexual decision making. Sociol Perspect.

[53] Shneyderman Y, Schwartz S (2013). Contextual and intrapersonal predictors of adolescent risky sexual behavior and outcomes. Heal Educ Behav.

[54] Siennick S (2013). Still the favorite? Parents’ differential treatment of siblings entering young adulthood. J Marriage Fam.

[55] Resnick M, Bearman P, Blum R (1997). Protecting adolescents from harm: findings from the National Longitudinal Study on adolescent health. JAMA..

[56] Volungis A (2016). School size and youth violence: the mediating role of school connectedness. N Am J Psychol.

[57] Williams A, Merten M (2014). Linking community, parenting, and depressive symptom trajectories: testing resilience models of adolescent agency based on race/ethnicity and gender. J Youth Adolesc..

[58] Crooks CV, Scott KL, Wolfe DA, Chiodo D, Killip S. Understanding the link between childhood maltreatment and violent delinquency: what do schools have to add? Child Maltreat. 2007. 10.1177/1077559507301843. 10.1177/1077559507301843 17631626

[59] Thornberry TP, Henry KL, Smith CA, Greenman SJ, Lee RD, Ireland TO (2013). Breaking the cycle of maltreatment: the role of safe, stable, and nurturing relationships. J Adolesc Health.

[60] Bender K. The mediating effect of school engagement in the relationship between youth maltreatment and juvenile delinquency. Child Sch. 2012. 10.1093/cs/cdr001.

[61] Craig J, Intravia J, Wolff K, Baglivio M (2017). What can help? Examining levels of substance (non) use as a protective factor in the effect of ACEs on crime. Youth Violence Juv Justice..

[62] Bronfenbrenner U (1981). The ecology of human development: experiments by nature and design.

[63] Folger SF, Wright MOD (2013). Altering risk following child maltreatment: family and friend support as protective factors. J Fam Violence.

[64] Brown SM, Shillington AM (2017). Childhood adversity and the risk of substance use and delinquency: the role of protective adult relationships. Child Abuse Negl.

[65] Fagan AA, Wright EM, Pinchevsky GM (2014). The protective effects of neighborhood collective efficacy on adolescent substance use and violence following exposure to violence. J Youth Adolesc..

[66] Komro KA, Flay BR, Biglan A, Consortium PNR (2011). Creating nurturing environments: a science-based framework for promoting child health and development within high-poverty neighborhoods. Clin Child Fam Psychol Rev.

[67] Blum R. School connectedness: improving the lives of students. Baltimore: Johns Hopkins Bloomberg School of Public Health; 2005. https://www.casciac.org/pdfs/SchoolConnectedness.pdf. Accessed 1 Apr 2018.

[68] Markowitz AJ (2017). Associations between school connection and depressive symptoms from adolescence through early adulthood: moderation by early adversity. J Res Adolesc.

[69] Chesmore AA, Weiler LM, Taussig HN (2017). Mentoring relationship quality and maltreated children’s coping. Am J Community Psychol.

[70] Kliewer W, Parrish KA, Taylor KW, Jackson K, Walker JM, Shivy VA (2006). Socialization of coping with community violence: influences of caregiver coaching, modeling, and family context. Child Dev.

[71] Holmes MR, Yoon S, Voith LA, Kobulsky JM, Steigerwald S (2015). Resilience in physically abused children: protective factors for aggression. Behav Sci (Basel, Switzerland).

[72] Meijers J, Harte M, Meynen G, Cuijpers P (2017). Differences in executive functioning between violent and non-violent offenders. Psychol Med.

[73] Trauffer N, Widom CS (2017). Child abuse and neglect, and psychiatric disorders in non-violent and violent female offenders. Violence Gend.

[74] Kalvin CB, Bierman KL (2017). Child and adolescent risk factors that differentially predict violent versus non-violent crime. Aggress Behav.

[75] Odgers CL, Moffitt TE, Tach LM (2009). The protective effects of neighborhood collective efficacy on British children growing up in deprivation: a developmental analysis. Dev Psychol.

[76] Browning CR, Gardner M, Maimon D, Brooks-Gunn J (2014). Collective efficacy and the contingent consequences of exposure to life-threatening violence. Dev Psychol.

[77] Manasseh T. We are reclaiming Chicago one corner at a time. The New York Times. https://www.nytimes.com/2017/10/22/opinion/chicago-gangs-crime-mothers.html. Published October 2017.

[78] Rees C, Pogarsky G (2011). One bad apple may not spoil the whole bunch: best friends and adolescent delinquency. J Quant Criminol.

[79] Rees C, Zimmerman GM (2016). The first delinquent peers are the most important: examining nonlinearity in the peer effect. Justice Q.

[80] Brauer JR, De Coster S (2015). Social relationships and delinquency: revisiting parent and peer influence during adolescence. Youth Soc.

[81] Chapple CL (2005). Self-control, peer relations, and delinquency. Justice Q.

[82] Siennick SE, Osgood DW (2012). Hanging out with which friends? Friendship-level predictors of unstructured and unsupervised socializing in adolescence. J Res Adolesc.

